# The NLR and LMR ratio in newly diagnosed MM patients treated upfront with novel agents

**DOI:** 10.1038/s41408-017-0019-6

**Published:** 2017-12-15

**Authors:** Alessandra Romano, Nunziatina Laura Parrinello, Claudio Cerchione, Maria Letizia Consoli, Marina Parisi, Valeria Calafiore, Enrica Martino, Concetta Conticello, Francesco Di Raimondo, Giuseppe Alberto Palumbo

**Affiliations:** 1 Department of Biomedicine and Molecular Medicine, University of Catania, Catania, Italy; 2 Division of Hematology, Azienda Policlinico-Vittorio Emanuele-Catania, Catania, Italy; 3 Hematology, Department of Clinical Medicine, University Federico II, Napoli, Italy

Multiple myeloma (MM) is the second most frequent hematological neoplasia, characterized by the accumulation of malignant plasma cells within the marrow microenvironment leading to variable anemia, bone pain, renal impairment, hypercalcemia and infections.

Virtually all cases of MM arise from monoclonal gammopathy of uncertain significance (MGUS), associated to a deep re-shape of the microenvironment and T-cell function. In MGUS, T-cells isolated from the bone marrow are able of mounting vigorous response against autologous pre-malignant cells while this phenomenon is not observed in MM^1^. Indeed, in MM the immune function is impaired as consequence of an immunologically hostile microenvironment and cellular defects^2^. MM plasma cells are able of immune editing through reduction of immune-surveillance, and expansion of myeloid derived suppressor cells as recently described in MM patients both at diagnosis and during chemotherapy^3, 4^. Several groups, including ours, identified NLR (the ratio between absolute neutrophils counts, ANC and absolute lymphocyte count, ALC) and LMR (the ratio between absolute lymphocyte counts, ALC and absolute monocyte count, AMC), as predictor of progression free survival (PFS) and overall survival (OS) in patients with hematological cancers^5, 6^, including MM^7–9^, as surrogate of a defective immune system. Several studies have searched for prognostic biomarkers before treatment start to choice the type and intensity of initial treatment. Recently, it has been proposed that the International Staging System should be associated to FISH results but the latter are not always available at diagnosis to address a tailored therapy^10^. We have shown that the combination of ISS with NLR is able to predict outcome in patients treated up-front with novel agents^8^. Indeed, NLR-ISS could identify patients that could benefit of single-novel agent based treatment and our results also confirm those recently published in another series that included patients treated with either novel agents (VMP, MPT) or older schemes (MP, VAD)^7^. It has published that NLR > 2 can be considered a bad prognostic factor for both PFS and OS in MM, as previously noticed in myeloma^8^ and lymphoma^6^.

We read with interest the analysis recently reported by Dosani et al. highlighting a LMR ratio < 3.6 as predictor of PFS and OS, also in patients with adverse cytogenetics, to stratify patients based on their baseline immune status^11^.

Thus, we reviewed files of 208 consecutively newly-diagnosed MM patients followed at our institution between January 2006 and June 2013, enrolled in observational or phase 3 clinical trials active in our Institutions (GIMEMA MMY-3006, RV-MM-PI209) for patients eligible to high-doses chemotherapy. Details on treatment regimens and final or ongoing results of these studies have previously been reported^8^. All studies were approved by our Institutional Review Board. Patients provided written informed consent before entering the studies, which were performed in accordance with the Declaration of Helsinki.

In all patients, complete blood count (CBC) and routine biochemical examinations were taken on every visit. White blood cell count and types (neutrophil, lymphocyte, eosinophil, and monocyte) were determined by electrical impedance method in automatic blood counter device (Beckman Coulter LH 750). NLR and LMR were calculated using data obtained from the CBC count. Baseline characteristics of evaluated patients are listed in Supplementary Table 1, based on NLR and LMR cut-offs respectively of 2 and 3.6 as previously published^8, 11^. Median age was 58 (range 31–66), 35% of patients were in stage III according to ISS classification. Cytogenetics was available for 199 (95%) patients, and it was adverse (del 17p or t (4;14)) in 13% of cases. Both high NLR and low LMR were associated to adverse FISH (Supplementary Table 1).

Induction regimens included bortezomib associated to dexamethasone (VD), thalidomide and dexamethasone associated or not to bortezomib (TD, VTD) accordingly to the GIMEMA MMY-3006 trial, or lenalidomide and dexamethasone, accordingly to the GIMEMA RV-MM-PI209^8^. Thus, 63% received bortezomib alone or in combination (23% in combination with IMiDs), 60% received lenalidomide or thalidomide alone or in combination, 95% patients underwent to single or double autologous stem cell transplantation as consolidation therapy. MM patients were divided in three groups based on the treatment received: regimen containing only proteasome inhibitor (group 1, *N* = 84) or IMiDs (group 2, *N* = 77) or both (group 3, *N* = 47). PFS was evaluated accordingly to Kaplan-Meier method. Descriptive statistics were generated for analysis of results and *p*-value under 0.05 was considered significant. Qualitative results were summarized in counts or percentages. Data were plotted as mean ± standard error mean or using boxes and whiskers at 5–95° percentile. Association among variables was evaluated by linear regression. Data were elaborated using GraphPad Prism version 6.00 for Windows, GraphPad Software, San Diego California USA, www.graphpad.com or MedCalc Version 12.3.0.0.

After a median follow up of 36 months, patients with NLR ≥ 2 had shorter PFS than patients with NLR < 2 (22.8 vs. 39.7 months, *p* = 0.025, Fig. 1a). Similarly, patients with LMR < 3.6 had shorter PFS than those with LMR ≥ 3.6 (18.5 vs. 40.5 months, *p* = 0.0003, Fig. 1b). Although ISS alone had a weak prognostic meaning in our series (*p* = 0.30, Fig. 1c), we tested if NLR or LMR could improve ISS.

**Fig. 1:**
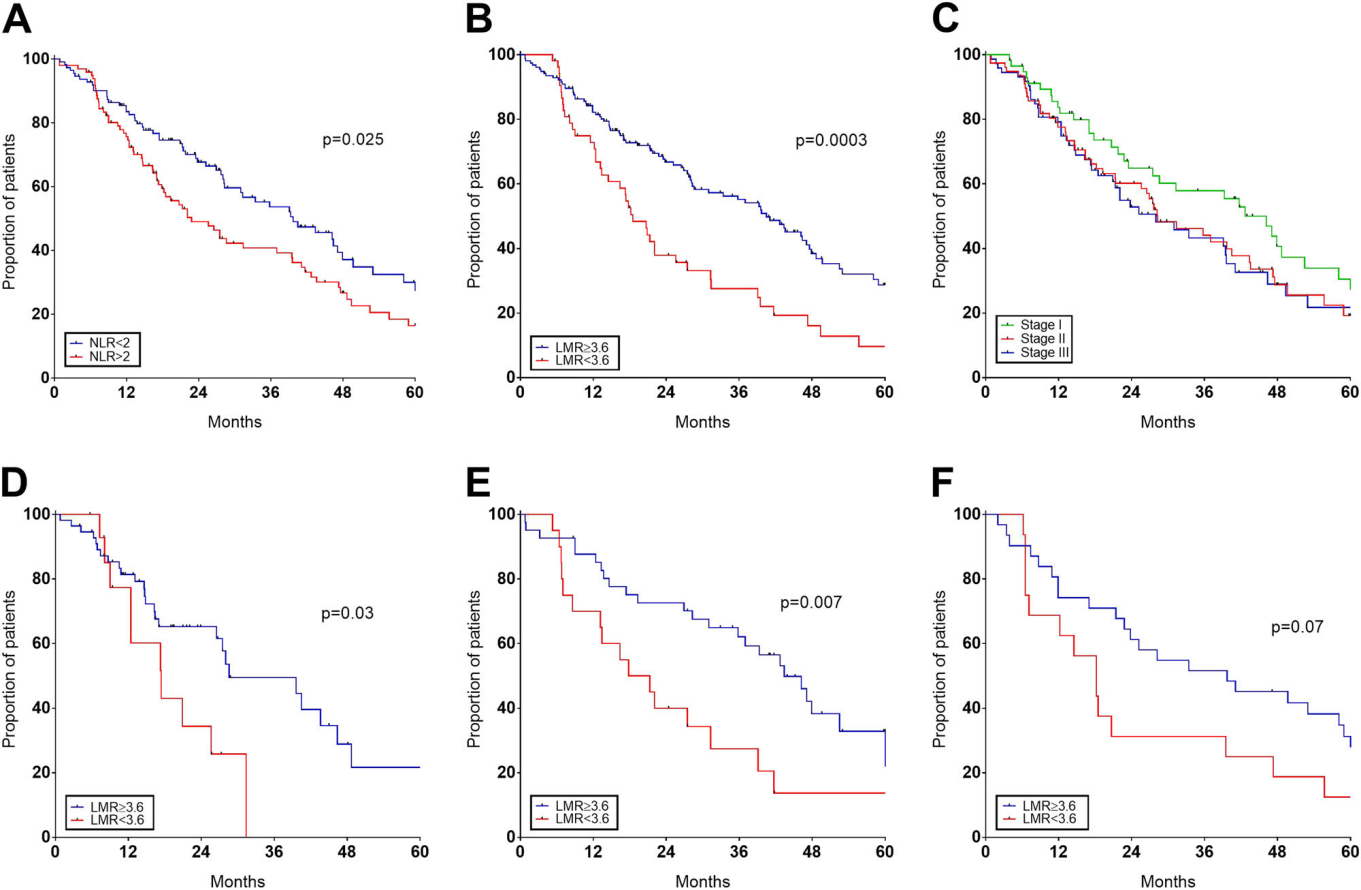
PFS in newly diagnosed MM treated upfront with novel agents PFS based on NLR **a**, LMR **b** or ISS **c** at diagnosis. PFS in the cohort of patients treated upfront with bortezomib-based **d**, lenalidomide- **e** or both **f** is shown based on LMR at diagnosis

As reported in Table 1, high NLR could discriminate prognosis in ISS stage I patients, while low LMR could predict inferior outcome in ISS-II/III patients. In multivariate analysis, predictors of PFS were LMR, ANC and absolute monocytes count as continuous variables (Supplementary Table 2), while LMR < 3.6 was independent from NLR.

**Table 1:** Progression free survival based on NLR, LMR, and ISS status

ISS	PFS in months	NLR status	*N*	PFS in months	*p*-value	LMR status	*N*	PFS in months	*p*-value
Stage 1 *N* = 54	46.2	NLR <2	27	64.3	*0.0003*	LMR >3.6	42	47.9	0.63
		NLR ≥2	27	20.7		LMR ≤3.6	12	27.5	
Stage 2 *N* = 77	28.3	NLR <2	40	28.3	0.71	LMR >3.6	53	37	*0.04*
		NLR ≥2	37	27.5		LMR ≤3.6	24	18.2	
Stage 3 *N* = 77	28	NLR <2	43	31	0.74	LMR >3.6	56	33.4	*0.012*
		NLR ≥2	34	22.1		LMR ≤3.6	21	18.5	

Since there was no difference in PFS in the three different treatment groups, and our previous work showed that NLR was predictor of outcome only in patients treated with lenalidomide or thalidomide, we tested if LMR was able to predict outcome independently from treatment used. Despite low numbers of this monocentric study, LMR < 3.6 was associated to inferior outcome in all groups of treatment, included the double combination of bortezomib and thalidomide (Fig. 1 d-f).

Our findings confirm the results of Dosani et al [22] and indicate that NLR and LMR could have a different biological meaning since they do not correlate each other and have a prognostic value in different subpopulation of patients. This difference is probably linked to the different role of neutrophils and monocytes in the complex network of the bone marrow microenvironment that supports myeloma growth and is further supported by the finding that neutrophils and monocytes counts are independent prognostic factors in multivariate analysis.

Thus, we confirm NLR and LMR as predictors of PFS in MM patients treated upfront with novel agents; this information could be integrated with FISH and molecular evaluations to personalise the treatment in younger patients. Patients with NLR ≥ 2 or LMR < 3.6 should be addressed to regimens containing both proteasome inhibitor and IMiDs. Integration of NLR and LMR to more detailed molecular data could result in a meaningful prognostic system that needs to be further validated.

## Electronic supplementary material

Supplemental Tables
