# Supplementary material for: The NLR and LMR ratio in newly diagnosed MM patients treated upfront with novel agents
Source: Blood Cancer J. 2017 Dec 15;7(12):649. doi: 10.1038/s41408-017-0019-6 (PMC5802528; doi:10.1038/s41408-017-0019-6)
Supplement: Supplementary file 1 — Supplemental Tables [file 41408_2017_19_MOESM1_ESM.docx]

**Supplementary Table 1**

**Patients’ characteristics**

|  | **NLR <2**  **N=110** | **NLR≥2**  **N=98** | **p-value** | **LMR ≥ 3.6**  **N=151** | **LMR<3.6**  **N=57** | **p-value** |
| --- | --- | --- | --- | --- | --- | --- |
| **Median age (range)** | 57 (49-6) | 60 (31-66) | 0.85 | 58 (49-66) | 59 (42-66) | 0.82 |
| **Males/Females** | 57/53 | 49/49 | 0.83 | 80/71 | 26/31 | 0.81 |
| **Monoclonal protein class, n (%)** |  |  |  |  |  |  |
| *IgG* | 60 (55) | 57 (58) | 0.77 | 83 (55) | 36 (63) | 0.61 |
| *IgA* | 38 (35) | 28 (29) | 0.82 | 44 (29) | 14 (25) | 0.32 |
| *Light chain only* | 12 (11) | 13 (13) | 0.81 | 24 (16) | 7 (12) | 0.11 |
| **Cytogenetics (FISH, IWMG criteria)** |  |  |  |  |  |  |
| Favorable | 60 (55) | 45 (46) | 0.20 | 85 (56) | 20 (35) | *0.02* |
| Intermediate | 39 (35) | 27(28) | 0.90 | 47 (31) | 19(33) | 0.31 |
| Adverse | 11 (10) | 17 (17) | *0.01* | 10 (7) | 18 (32) | *<0.001* |
| **WBC^.^1000/uL (range)** | 5.7 (4.4-10.5) | 7.3 (4.7-9.5) | *<0.001* | 6.1 (4.8-10.3) | 7.4 (4.4-10.2) | *<0.001* |
| **ANC^.^1000/uL (range)** | 2.7 (0.4-7.3) | 5.3 (1.2-21.3) | *<0.001* | 3.4 (0.4-21.3) | 5.3 (0.8-16.3) | *<0.001* |
| **AMC^.^1000/uL (range)** | 0.4 (0.1-1.3) | 0.5 (0.1-1.7) | 0.15 | 0.4 (0.1-1.7) | 0.6 (0.1-1.7) | *0.01* |
| **ALC^.^1000/uL (range)** | 2.1 (0.3-4.2) | 1.4 (1.0-3.2) | 0.82 | 2.1 (0.3-4.2) | 1.9 (1.0-3.8) | 0.83 |
| **Plasma cells infiltrate % (range)** | 54 (30-100) | 52 (30-100) | 0.88 | 34 (40-90) | 54 (30-100) | 0.16 |
| **LDH, U/L (range)** | 254 (103-215) | 279 (109-708) | 0.87 | 256 (103-708) | 278 (136-708) | 0.86 |
| **Beta-2 microglobulin, mg/L (range)** | 3.9 (1.8-3.2) | 4.1 (1.9-9.9) | 0.82 | 2.8 (1.8-3.2) | 4.1 (1.9-9.9) | 0.65 |
| **Serum Albumin, g/dL (range)** | 3.7 (3.2-4.2) | 3.6 (2.5-4.5) | 0.81 | 3.9 (3.2-4.2) | 3.9 (2.5-4.5) | 0.82 |
| **STAGE ISS (%)** |  |  |  |  |  |  |
| *I* | 27 (25) | 27 (28) | 0.82 | 42 (28) | 12 (21) | 0.34 |
| *II* | 40 (36) | 37 (38) | 0.82 | 53 (35) | 24 (42) | 0.16 |
| *III* | 43 (39) | 34 (35) | 0.82 | 56 (37) | 21 (37) | 0.82 |

**Suplementary Table 2**

**Multivariate analysis of progression free survival**

| **Covariate** | **b** | **SE** | **p-value** | **Exp(b)** | **95% CI of Exp(b)** |
| --- | --- | --- | --- | --- | --- |
| **ISS** | 0.19 | 0.13 | 0.13 | 1.21 | 0.94 to 1.54 |
| **NLR** | -0.01 | 0.06 | 0.83 | 0.99 | 0.87 to 1.10 |
| **LMR** | 0.11 | 0.05 | *0.03* | 1.11 | 1.01 to 1.22 |
| **ANC** | 0.00 | 0.00 | *0.01* | 1.00 | 0.99 to 0.99 |
| **ALC** | 0.00 | 0.00 | 0.32 | 1.00 | 0.99 to 1.00 |
| **AMC** | 0.00 | 0.00 | *0.02* | 1.00 | 1.00 to 1.01 |
| **LMR <3.6** | 0.56 | 0.22 | *0.01* | 1.76 | 1.14 to 2.69 |
| **NLR ≥ 2** | 0.25 | 0.21 | 0.23 | 1.29 | 0.85 to 1.93 |
